# Predicting the formation of different tissue types during Achilles tendon healing using mechanoregulated and oxygen-regulated frameworks

**DOI:** 10.1007/s10237-022-01672-4

**Published:** 2022-12-21

**Authors:** Thomas Notermans, Hanna Isaksson

**Affiliations:** Department of Biomedical Engineering, Lund University, Lund, Sweden

**Keywords:** Mechanobiology, Angiogenesis, Endochondral ossification, Heterotopic ossification, Cell infiltration

## Abstract

During Achilles tendon healing in rodents, besides the expected tendon tissue, also cartilage-, bone- and fat-like tissue features have been observed during the first twenty weeks of healing. Several studies have hypothesized that mechanical loading may play a key role in the formation of different tissue types during healing. We recently developed a computational mechanobiological framework to predict tendon tissue production, organization and mechanical properties during tendon healing. In the current study, we aimed to explore possible mechanobiological related mechanisms underlying formation of other tissue types than tendon tissue during tendon healing. To achieve this, we further developed our recent framework to predict formation of different tissue types, based on mechanobiological models established in other fields, which have earlier not been applied to study tendon healing. We explored a wide range of biophysical stimuli, i.e., principal strain, hydrostatic stress, pore pressure, octahedral shear strain, fluid flow, angiogenesis and oxygen concentration, that may promote the formation of different tissue types. The numerical framework predicted spatiotemporal formation of tendon-, cartilage-, bone- and to a lesser degree fat-like tissue throughout the first twenty weeks of healing, similar to recent experimental reports. Specific features of experimental data were captured by different biophysical stimuli. Our modeling approach showed that mechanobiology may play a role in governing the formation of different tissue types that have been experimentally observed during tendon healing. This study provides a numerical tool that can contribute to a better understanding of tendon mechanobiology during healing. Developing these tools can ultimately lead to development of better rehabilitation regimens that stimulate tendon healing and prevent unwanted formation of cartilage-, fat- and bone-like tissues.

**Supplementary Information:**

The online version contains supplementary material available at 10.1007/s10237-022-01672-4.

## Introduction

**Fig. 1:**
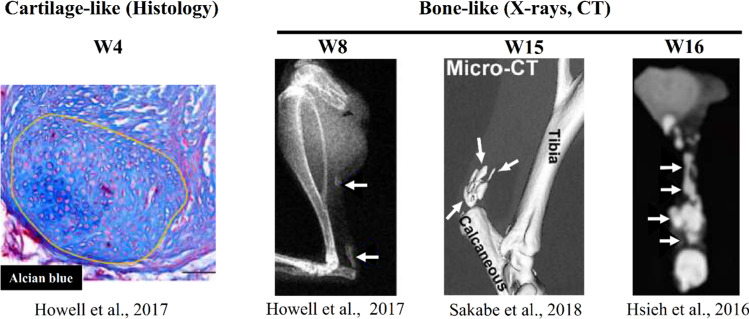
Examples of literature evidence that tendon healing involves formation of cartilage-like and bone-like tissues. The circled area in the histological section (left) with Alcian blue depicts cartilage deposition and cell morphology. The X-ray and CT images (right) depict the high-density bone-like tissue formation (white arrows). Images were collected from the following references (Howell et al. 2017; Hsieh et al. 2016; Sakabe et al. 2018), reused under the Creative Commons CC-BY and Creative Commons Attribution license 4.0. More literature examples of cartilage-like and bone-like tissue formations throughout 17 weeks of healing in rat Achilles tendon can be found in: (Asai et al. 2014; da Silva et al. 2020; Howell et al. 2017; Hsieh et al. 2016; Huber et al. 2020; Lin et al. 2010; Misir et al. 2019; Sakabe et al. 2018)

The incidence of Achilles tendon rupture has been increasing throughout the last decades (Ganestam et al. 2016; Huttunen et al. 2012; Lemme et al. 2018; Nyyssönen et al. 2008). The rehabilitation regimen after rupture could play a key role for the healing outcome (Holm et al. 2015), where, e.g., different mechanical loading regimens have been found to affect the outcome in humans (El-Akkawi et al. 2018; Ochen et al. 2019). To design loading protocols that better stimulate tendon healing, there is a need to understand tendon mechanobiology, i.e., the adaptation of tendon properties to external mechanical loading. To study this, small animal models are most commonly used (Notermans et al. 2021a). Recent experimental studies in rodents have reported significant aberrant formation of tissues other than tendon tissue, e.g., formation of fat- (Huber et al. 2020; Khayyeri et al. 2020), cartilage- (Asai et al. 2014; da Silva et al. 2020; Howell et al. 2017; Khayyeri et al. 2020; Korntner et al. 2017; Misir et al. 2019) or bone-like tissue features (Asai et al. 2014; Chen et al. 2017; Hsieh et al. 2016; Huber et al. 2020; Lin et al. 2010; Sakabe et al. 2018; Zhang et al. 2016) (Fig. 1). These studies showed that areas of cartilage-like tissue could be identified after between four and 17 weeks of healing, whereas bone-like tissue was present from five up to 16 weeks of healing (Lin et al. 2010) (Fig. 1). Particularly, the long-term studies showed that bone-like tissue may take up a large volume of the healing tendon at the later time points (Hsieh et al. 2016; Sakabe et al. 2018).

Several studies have found fat-, cartilage- (Khayyeri et al. 2020) and bone-like cells (Lin et al. 2010) throughout early healing. In particular, chondrocyte-like cells were identified already in the first weeks of healing (Asai et al. 2014; da Silva et al. 2020; Khayyeri et al. 2020). Asai et al. (2014) found cartilage-like cells (round cells) between two to four weeks, they observed cartilage-specific matrix proteins (collagen type 2 and aggrecan) at eight weeks, and they found bone-like tissue at 12 weeks in all rat Achilles tendons in their study using CT imaging. Santos Da Silva et al. (2020) also observed progressive production of collagen type 2 throughout 17 weeks of healing, and chondrocyte-like cells were still present around islands of mineralized tissue at 17 weeks. Howell et al. (2017) hypothesized that tenocytes (intrinsic tendon cells) contribute to cartilage formation in the tendon stumps and that this may be a key factor why the tendon heals poorly. Another study pointed out that cartilage could facilitate bone formation through the endochondral pathway (Lin et al. 2010). They showed that an initial chondrogenic phase was followed by bone formation, which started between three and five weeks of healing. The chondrogenic phase also displayed high expression of hypoxia-inducible factor 1α, whereas the bone formation phase displayed high vascular endothelial growth factor expression. This indicates that cartilage formation may occur during hypoxic conditions, whereas bone formation typically occurs in presence of blood vessels (providing sufficient oxygen). This complies well with what is known from other regenerative situations in experimental (e.g., Buckley et al. 2010; Hausman et al. 2001; Hirao et al. 2006)) and numerical studies (e.g., Burke and Kelly 2012; Checa et al. 2010; Geris et al. 2008)). Another study in rotator cuff tendons showed that calcifications were surrounded by blood vessels (Darrieutort-Laffite et al. 2019), confirming that angiogenesis and oxygen levels play important roles in bone-like tissue formation in tendons as well.

Mechanobiological review articles on tendon healing mentioned that mechanical loading, in particular over- and unloading, may cause aberrant tissue formation (Freedman et al. 2015; Notermans et al. 2021a). It has been reported that the level of bone-like tissue formation during healing depends on the level of loading on the tendon. Recently, Huber et al. (2020) showed that joint immobilization could limit bone-like tissue formation (Huber et al. 2020). The authors proposed that joint immobilization was associated with decreased collagen organization, cell spreading and transcriptional activator with PDZ-binding domain (TAZ) signaling, thereby inducing adipocyte differentiation. Oppositely, they proposed that fiber alignment, cell spreading and TAZ signaling increases upon loading, inducing ectopic bone formation. The bone-like tissue volume after six weeks of healing was highest for the loaded tendon experiencing free cage activity (Huber et al. 2020). However, partial immobilization decreased the amount of fibrocartilage (Palmes et al. 2002) and bone-like tissue volume (Chen et al. 2017), compared to full immobilization.

Although experimental evidence is accumulating, there is no computational framework to date that has investigated mechanoregulated tissue differentiation or the formation of different tissue types during tendon healing. However, there is a range of numerical algorithms available that investigated the role of mechanical loading during bone regeneration (Burke and Kelly 2012; Carter et al. 1998; Checa et al. 2010; Claes and Heigele 1999; Isaksson et al. 2008; Lacroix and Prendergast 2002). A wide range of biophysical stimuli has been explored, i.e., principal or octahedral shear strain, pore pressure, hydrostatic stress or fluid flow, in terms of its ability to regulate cell differentiation and subsequent formation of different tissue types, e.g., cartilage, (im)mature bone, bone marrow, granulation tissue and fibrous tissue (Isaksson et al. 2008, 2006b). These finite element frameworks were later expanded, for example, by investigating the role of mechanoregulated angiogenesis, local tissue stiffness and oxygen concentration in terms of its effect on tissue differentiation during bone healing (Burke and Kelly 2012).

We recently developed a mechanobiological tendon healing framework that incorporates mechanical and cellular regulatory mechanisms to predict spatial and temporal tendon tissue production, organization and mechanical properties (Notermans et al. 2022, 2021b). In the current study, we aimed to explore possible mechanobiological mechanisms underlying the formation of other tissue types during tendon healing. To investigate this, we further developed our recent framework (Notermans et al. 2022, 2021b) to include predictions of tissue differentiation and subsequent formation of different tissue types, based on knowledge from the field of bone regeneration (Burke and Kelly 2012; Carter et al. 1998; Claes and Heigele 1999; Isaksson et al. 2006b; Lacroix and Prendergast 2002). The presented framework is able to capture heterogeneous production of tendon-, cartilage- and bone-like tissues throughout tendon healing. The predictions are compared to qualitative observations in recent experimental studies.

## Methods

**Fig. 2:**
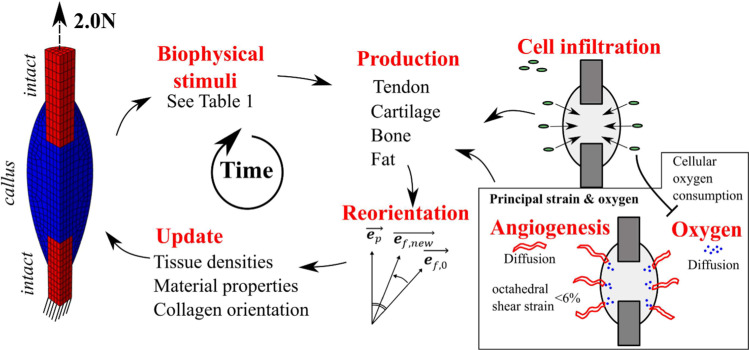
Overview of the iterative framework to predict formation of different tissue types during tendon healing. A 2 N mechanical load was applied to the FE model and a wide range of biophysical stimuli were calculated according to existing tissue differentiation algorithms (see Table 1) to predict formation of tendon-, cartilage-, bone- and fat-like tissues. Diffusion simulations were utilized to model cell infiltration, angiogenesis and oxygen diffusion, where oxygen was consumed by cells

We recently developed a mechanobiological framework that allows us to predict tendon tissue formation and reorientation in response to mechanical stimulation (Notermans et al. 2022, 2021b). Briefly, a 3D finite element model for tendon healing was combined with an existing fiber-reinforced hyper-visco-poro-elastic material model tendon (Khayyeri et al. 2016; Notermans et al. 2019). An iterative framework (Fig. 2) was implemented to predict spatial and temporal tissue production, collagen reorientation and the temporal evolution of mechanical properties in the healing tendon callus (see more details in Notermans et al. 2022, 2021b)). This was used as a starting point in the current study. In the current study, the tendon was stimulated with a mechanical load during every iteration of healing, and subsequently, tendon-, cartilage-, bone- or fat-like tissue was predicted to form in the healing callus, depending on a range of different biophysical stimuli (Table 1). In addition, the process of endochondral bone formation was explored, and a parameter sensitivity analysis of the angiogenesis and oxygen-dependent framework was performed.

**Table 1:** Overview of the biophysical thresholds implemented to predict formation of tendon, fat, cartilage- and bone-like tissues

Stimuli:	Princ. strain	Princ. Strain & Hydro. stress	Princ. Strain & pore pressure	Octa. shear strain & Fluid flow	Princ. Strain & oxygen angiogenesis
Model:	PE	PE-HS	PE-PP	OSS-FF	PE-OXY (A)
Reference:		(Carter et al. 1998)	(Claes and Heigele 1999)	(Lacroix and Prendergast 2002)	(Burke and Kelly 2012)
Tendon	> 4*	> 5; < 0.2	> 15; > 0.15 > 5; < − 0.15 or > 0.15	stim > 3	–;3; < 90* 2–25%; > 3; > 90*
Cartilage	2–4	< 5; > 0.2	< 15; > 0.15	1 < stim < 3	–; < 3; –
Bone	< 2	< 5; < 0.2	< 5; < ± 0.15	stim > 1	< 2; –; > 90
Fat	–	–	–	–	> 25;–; > 90

Different biophysical stimuli, i.e., maximum principal strain (PE, %), hydrostatic stress (HS, MPa), pore pressure (PP, MPa), octahedral shear strain (OSS, %), fluid flow (FF, μm/s), oxygen concentration (OXY, %) and angiogenesis (A, %), were based on (Burke and Kelly 2012; Carter et al. 1998; Claes and Heigele 1999; Lacroix and Prendergast 2002). The octahedral shear strain and fluid flow algorithm is based on a general stimulus (stim) that is calculated according: stim = OSS/3.75 + FF/3 (Lacroix and Prendergast 2002). The (maximum) principal strain (PE) and principal strain with oxygen algorithms included tendon production (noted with asterisks) according to a strain magnitude-dependent production law (Online Resource 4) utilized in a previous study when predicting strain-dependent tendon formation (Notermans et al. 2022, 2021b)

### The finite element model

A finite element (FE) mesh was created based on geometrical measurements from healing rat Achilles tendon that had been subjected to 1 week of free cage activity after rupture (details on geometry and boundary conditions are available in Online Resource 1 and 2) (Khayyeri et al. 2020). The healing tendon consisted of two stumps with aligned collagen fibers (one direction, anisotropic) and a bulging healing callus with 13 collagen fiber directions (simulating random orientation) in every material point. The densities of collagen and ground matrix in the callus were set to 10% (compared to stumps) at the start of healing. The FE model was subjected to 2.0 N tensile load, representing the maximum force during gait in adult female Sprague–Dawley rats (Song et al. 2019). Mechanical loading was applied as a linear ramp at a rate of 1.1 N/s. All simulations were performed in Abaqus v2017 (Dassault Systèmes Simulia Corp., Johnston, RI, USA).

### Mechanoregulatory schemes

Mechanoregulatory algorithms based on different biophysical stimuli were used to predict the tissue formation of tendon-, fat-, cartilage- and bone-like tissue (Table 1). Several existing mechanoregulatory schemes were adopted and investigated (Carter et al. 1998; Claes and Heigele 1999; Lacroix and Prendergast 2002). These algorithms utilize hydrostatic stress (HS) and octahedral shear strains (OSS) that were calculated according to:

$$\begin{aligned} \sigma_{{{\text{hydrostatic}}}} & = \frac{{{\text{tr}}\left( {\varvec{\sigma}} \right)}}{3} = \frac{{(\sigma_{1} + \sigma_{2} + \sigma_{3} )}}{3} \\ \varepsilon_{{{\text{os}}}} & = \frac{1}{3}\sqrt {\left( {{\upvarepsilon }_{1} - {\upvarepsilon }_{3} } \right)^{2} + \left( {{\upvarepsilon }_{1} - {\upvarepsilon }_{2} } \right)^{2} + \left( {{\upvarepsilon }_{2} - {\upvarepsilon }_{3} } \right)^{2} } \\ \end{aligned}$$

where hydrostatic stress ($\sigma_{{{\text{hydrostatic}}}}$) is defined using the trace of the stress tensor (${\varvec{\sigma}}$) in Cartesian format and octahedral shear strain ($\varepsilon_{os}$) depends on the maximum (${\upvarepsilon }_{1}$), mid- (${\upvarepsilon }_{2}$), and minimum (${\upvarepsilon }_{3}$) principal strains. In addition to these existing algorithms, a new mechanoregulatory scheme was designed using solely the (maximum) principal strain (PE) thresholds for predicting cartilage- (2–4%) and bone-like tissue formation (< 2%). Simulations of intact tendon were performed to verify the validity of these thresholds (Online Resource 3). The same strain thresholds were utilized in combination with the oxygen framework by Burke et al. (2012), which was originally designed using local matrix stiffness instead of principal strain. A principal strain threshold for fat (> 25%) was added to the oxygen framework. The 3% oxygen concentration and 90% angiogenesis threshold for cartilage- and bone-like tissue formation, respectively, were adopted from the study by Burke and Kelly (2012).

### Tissue production rates

The healing framework describing collagen production and reorientation laws and rates and cell infiltration were implemented as described earlier (Notermans et al. 2022). Tendon-, cartilage- and fat-like tissue were produced at the default rate (2%/day), whereas bone-like tissue was produced at 1.2%/day (similar to the implementation in Isaksson et al. (2006a)). Tendon production in the mechanoregulatory algorithms based on principal strain with (PE-OXY) or without oxygen (PE) was based on a strain-regulated production law (Online Resource 4) (Notermans et al. 2022, 2021b) that predicts an initial increase in the tissue production with increasing strain. However, for principal strains over 15%, tissue production decreases with increasing strain. For all models, during the first five days of healing, a baseline tissue production rate (50% of daily production) was assumed to be driven by acute inflammation. After 5 days, the tissue production rate was solely mechanoregulated (Notermans et al. 2021b). For all models, cell infiltration from the extrinsic compartment of the callus was considered (Notermans et al. 2022) (Fig. 2; Online Resource 1 and 5). The cell infiltration rate was set to reach 95% cell density after 2 weeks. The local tissue production in an element in the callus was linearly dependent on the local cell density such that no tissue production occurs if there are no cells present, regardless of the mechanical cue, and mechanoregulated tissue production is allowed fully if the local cell density is 100%. Degradation of tissue was also considered in all models. Namely, as one tissue type is produced, other tissues are degraded at the production rate of the tissue type that is produced.

### Material properties for different tissues

To describe the material properties of fat-, tendon-, cartilage- and bone-like tissue, a tissue type-dependent material behavior was implemented. Scaling coefficients were used to adapt the material behavior for the different tissue types compared to tendon material properties that were determined in intact tendon (Notermans et al. 2019). The scaling coefficient for fat tissue (0.5) was implemented to ensure a decrease in stiffness for fat-like tissue, compared to tendon-like tissue, following Burke et al. (2012). The scaling coefficients for cartilage- and bone-like tissue (2.62, 40.40) were implemented to ensure that the cartilage- and bone-like tissue are 50 and 500 times stiffer than tendon tissue at 2 N load according to an earlier computational framework for predicting tissue differentiation and formation in bone healing (Isaksson et al. 2006a). The material properties were then implemented according to:

$$M_{{{\text{tissue}}}}^{{{\text{Callus}}}} = (0.5*\rho^{F} + \rho^{T} + \rho^{C} *2.62 + \rho^{B} *40.40)*M_{{{\text{Tendon}}}}^{{{\text{intact}}}} \;for\;M = E_{1} ,E_{2} ,K_{1} ,K_{2} ,\; E_{p} , E_{n} ,G_{{{\text{pn}}}}$$

where the material parameter (M) in the healing callus depends on the local fat ($\rho^{F}$), tendon ($\rho^{T}$), cartilage ($\rho^{C}$) and bone ($\rho^{B}$) density. We scaled all stiffness parameters in our constitutive material model (Khayyeri et al. 2016; Notermans et al. 2019), for both the collagen ($E_{1} ,E_{2} ,K_{1} ,K_{2}$*)* and ground substance ($E_{p} , E_{n} ,G_{pn}$).

### Reorientation

In each iteration of the healing framework, the collagen fibrils (13/material point with random initial orientation) in the callus were rotated in the direction of the maximum principal strain (Notermans et al. 2022, 2021b; Tanska et al. 2018; Wilson et al. 2006). The fibril reorientation from random to longitudinal alignment was set to occur in four weeks (Notermans et al. 2022, 2021b).

### Endochondral bone formation

The different mechanobiological algorithms assumed that bone-like tissue formation depends on local mechanical stimuli or the presence of blood supply. In addition to these requirements, the effect of limiting bone formation to endochondral bone formation was investigated (Lin et al. 2010), i.e., that bone can only form through ossification of cartilage or further ossification/apposition of existing bone. This process was investigated using the principal strain model (referred to as PE-ENDO). The implementation limited bone-like tissue formation to only occur if the two following requirements were met:

- (Maximum) principal strain < 2%,
- Current density of cartilage ($\rho^{C} > \rho_{{{\text{endo}}}}^{C}$) OR bone ($\rho^{B} > 0\%$) density,

where two different threshold values for the cartilage density ($\rho_{{{\text{endo}}}}^{C} =$ 20 or 25%) were explored (referred to as PE-ENDO 20% or PE-ENDO 25%).

### Angiogenesis and oxygen framework

Diffusion simulations for angiogenesis and oxygen were performed every iteration of healing, similarly to an existing framework for oxygen-dependent bone healing (Burke and Kelly 2012). At the first iteration of healing, the callus was deprived of blood vessels and oxygen (angiogenesis and oxygen density was 0%). Every iteration of the healing framework (~ 1 day), angiogenesis and oxygen diffusion was allowed to occur from the external surface into the healing callus (Online Resource 1 and 5) Angiogenesis and oxygen diffusion were modeled using Darcy’s law for diffusion according to:

$\frac{{{\text{d}}\rho }}{{{\text{d}}t}} = D\nabla^{2} \rho$ with diffusion constant ($D$) and density ($\rho$) (Burke and Kelly 2012; Isaksson et al. 2008). Angiogenesis then occurred in elements where the average octahedral shear strain was lower than a threshold value (A-OSS) (Burke and Kelly 2012; Simon et al. 2011). A node with more than 90% angiogenesis was considered a matured blood vessel that provided blood supply, and thus, this node was a new source for oxygen. In addition, bone-, fat- and tendon-like tissue were allowed to form at these established blood supplies. Additionally, cells were able to consume oxygen (maximum 50% oxygen was consumed at 100% cell density, C = 0.5) according to:

$$\frac{{{\text{d}}\rho^{{{\text{oxygen}}}} }}{{{\text{d}}t}} = O\nabla^{2} \rho^{{{\text{oxygen}}}} {-}C*\rho^{{{\text{cells}}}} *\rho^{{{\text{oxygen}}}}$$

with the diffusion constant ($O$), oxygen density ($\rho^{{{\text{oxygen}}}}$), tuning parameter for cell-dependent oxygen diffusion (C) and cell density ($\rho^{{{\text{cells}}}}$). The predicted angiogenesis and oxygen distributions affected the tissue formation according to Table 1. To determine how sensitive the predicted tissue distributions were with regards to different angiogenesis- and oxygen-related parameters, a parameter sensitivity analysis was performed, varying the diffusion constant for angiogenesis (A = 0.25–0.5–1.0) and oxygen (O = 0.25–0.5–1.0), the extent of cellular oxygen consumption (C = 0.25–0.5–0.75) and the octahedral shear strain threshold for angiogenesis (A-OSS = 3–6–12%).

### Healing predictions

**Fig. 3:**
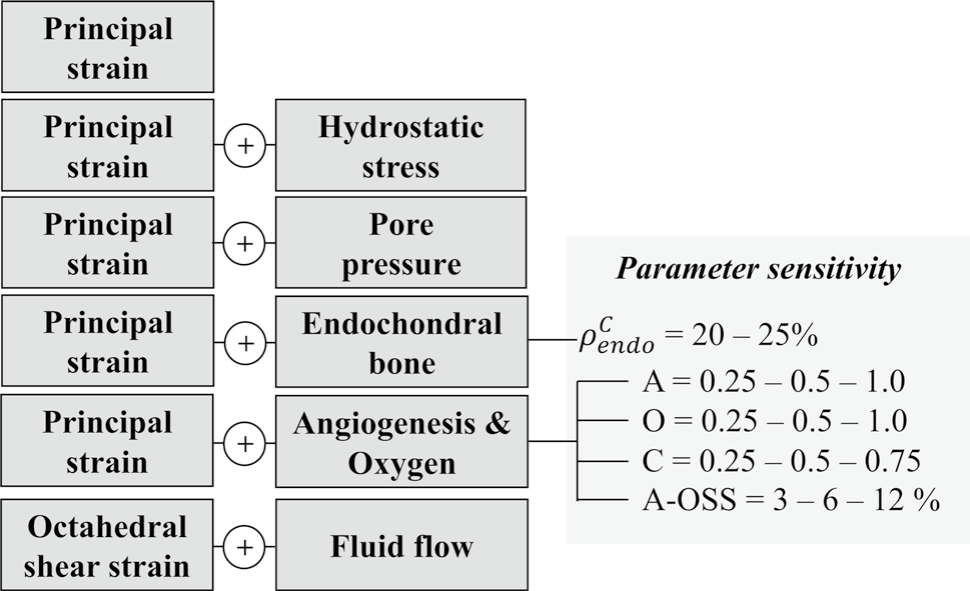
Overview of the six different mechanoregulatory frameworks investigated in this study and an overview of the parameter sensitivity analysis for the threshold of endochondral bone formation (${\uprho }_{{{\text{endo}}}}^{{\text{C}}}$), diffusion constants for simulations of angiogenesis (A), oxygen diffusion (O) and cell-dependent oxygen consumption O. The different algorithms are referred to with these abbreviations: principal strain (PE), principal strain and hydrostatic stress (PE-HS), principal strain and pore pressure (PE-PP), principal strain and oxygen (PE-OXY), principal strain with endochondral bone formation (PE-ENDO), octahedral shear strain and fluid flow (OSS-FF)

A total of six mechanobiological algorithms were investigated (Fig. 3). From each simulation, the predicted temporal and spatiotemporal evolution of tendon, fat-, cartilage- and bone-like tissue density was characterized throughout 20 weeks of tendon healing and compared to a range of literature findings. Furthermore, the temporal evolution of stiffness at 2 N was calculated and compared to experimental data from intact (Khayyeri et al. 2017) and healing (Khayyeri et al. 2020) rat Achilles tendon. Predicted bone tissue formation was validated against in-house measurements of bone-like tissue during rat Achilles tendon healing (Pierantoni et al. 2022). To determine the absolute volumes of bone-like tissue throughout healing, tissue volumes were segmented from 3D tomography data (phase contrast-enhanced X-ray microtomography at the Diamond-Manchester Imaging Branchline I13–2) from healing rat Achilles tendon at 1, 3, 12 and 20 weeks of healing (*n* = 3 at each time point). The volume of interest for this quantification was the whole tendon, including both the healing callus and the tendon stumps. To determine the bone-like tissue volume in the simulations, we integrated the element-level bone density multiplied by element volume, for all elements in the callus.

## Results

This section presents the results of all mechanobiological mechanisms presented in Table 1 (PE, PE-HS, PE-PP, PE-OXY, OSS-FF), and the results predicted when limiting bone formation to the endochondral pathway (PE-ENDO) compared to the default strain model (PE). Finally, the parameter analysis of the strain- and oxygen-dependent algorithm (PE-OXY) is presented.

### Temporal evolution of tissue formation and mechanical properties

**Fig. 4:**
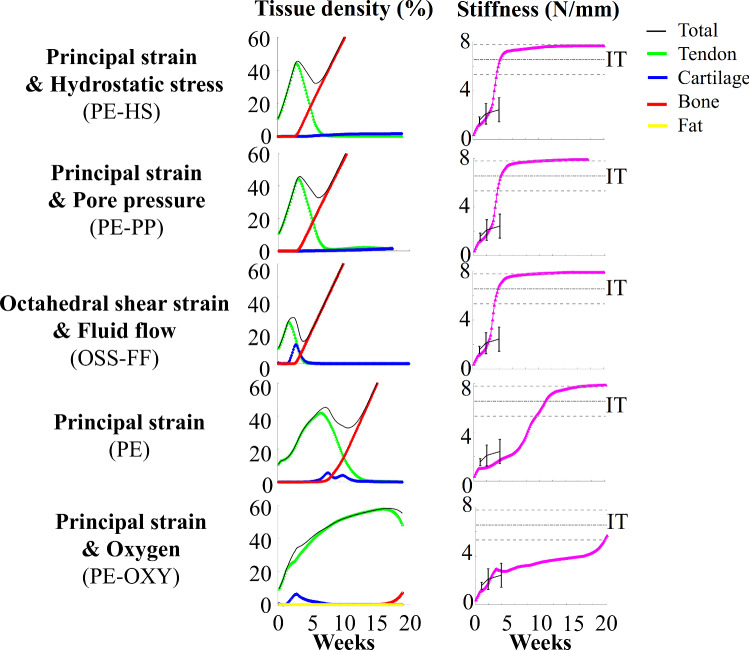
Temporal evolution of tendon-, cartilage-, fat- and bone-like density and stiffness throughout 20 weeks of healing. Stiffness data are compared to experimental data (black lines, mean ± standard deviation) from intact (IT) (Khayyeri et al. 2017) and healing rat Achilles tendons subjected to free cage activity loading at 1, 2 and 4 weeks (Khayyeri et al. 2020)

The implemented mechanobiological algorithms predicted a unique sequential evolution of tendon-, cartilage- and bone-like tissue formation throughout the first 20 weeks of healing (Fig. 4). All algorithms predicted formation of tendon tissue initially and ended with predicting a significant amount of bone-like tissue formation. The octahedral shear strain and fluid flow stimulus (OSS-FF) predicted a shorter tendon production phase and earlier prediction of bone-like tissue formation. On the other hand, the principal strain and oxygen stimulus (PE-OXY) predicted formation of tendon-like tissue for a longer time period. It was also the latest to predict formation of bone-like tissue.

Cartilage-like tissue formation was less prominent than the formation of tendon- or bone-like tissue. Principal strain combined with hydrostatic stress (PE-HS) or pore pressure (PE-PP) predicted slow but gradual cartilage-like tissue production that persisted throughout 20 weeks of healing. On the other hand, the other biophysical stimuli (PE, PE-OXY, OSS-FF) predicted cartilage-like tissue production over a short time span. No fat production was predicted by the principal strain and oxygen stimulus (PE-OXY).

With the progressive formation of tendon-, cartilage- and bone-like tissue, the stiffness of the healing tendon increased throughout healing (Fig. 4). The principal strain and oxygen stimulus (PE-OXY) predicted the latest onset of bone-like tissue formation, and therefore, it also predicted the slowest stiffness evolution. Yet, the predicted stiffness in this model was within the range of the experimental data during the first weeks of healing and reached intact levels of stiffness at 20 weeks of healing. The other algorithms (PE, PE-HS, PE-PP, OSS-FF) predicted that the stiffness would reach intact levels earlier, i.e., after four to twelve weeks of healing. After reaching intact level of stiffness, the stiffness evolution flattened in an asymptotic fashion.

### Spatial evolution of tissue formation

**Fig. 5:**
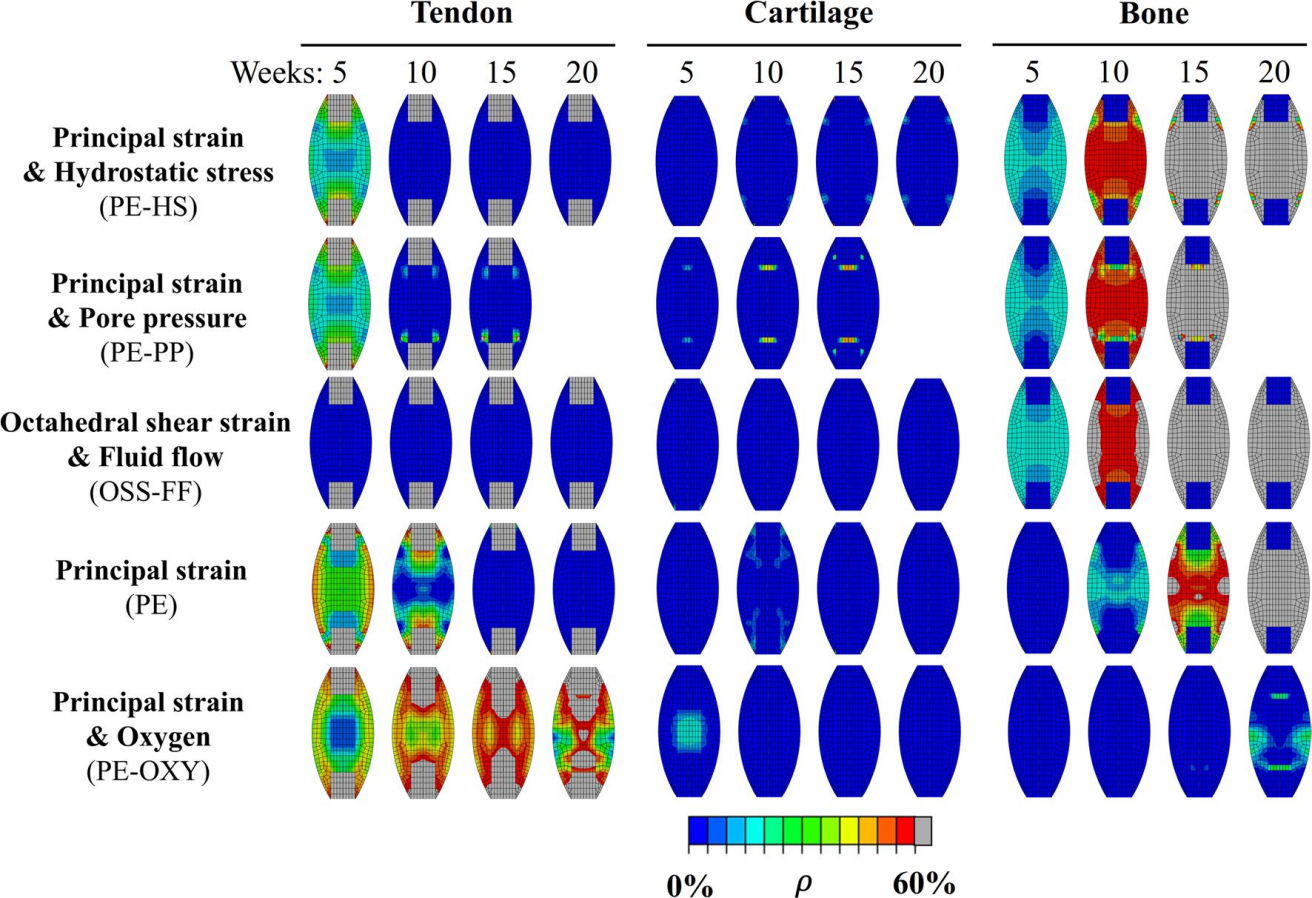
Spatiotemporal evolution of tendon-, cartilage- and bone-like tissue density (*ρ*) at 5, 10, 15 and 20 weeks of healing

All mechanobiological algorithms predicted heterogeneous tissue formation throughout 20 weeks of healing (Fig. 5). During the initial phase of tendon formation, the production was initially higher in the callus periphery compared to the callus core. This was followed by a homogeneous production of cartilage-like and bone-like tissue. The principal strain and oxygen stimulus (PE-OXY) predicted the longest production of tendon tissue. This algorithm also showed the most heterogeneous tendon formation with high tendon content in the periphery for at least 10 weeks. Tendon density disappeared quickest in the simulations with octahedral shear strain and fluid flow (OSS-FF) as the regulatory stimulus.

Also, the spatial mapping of the density evolution display that cartilage-like tissue production is less prominent than either tendon- or bone-like tissue production for all stimuli. Yet, principal strain and oxygen (PE-OXY) predicted the highest content of cartilage-like tissue in the callus core. On the other hand, the principal strain and pore pressure (PE-PP) stimulus predicted long-term cartilage-like tissue production at the stump interface. Principal strain alone (PE) or with hydrostatic stress (PE-HS) predicted small regions of cartilage-like tissue production next to the tendon stumps in the periphery.

As mentioned above, all mechanobiological algorithms predicted formation of bone-like tissue. Bone-like tissue was generally first formed in the periphery of the callus, before spreading to the callus core and throughout the whole callus. Yet, for the principal strain and oxygen stimulus (PE-OXY), bone-like tissue formation was predicted rather late and did not spread to the whole callus by 20 weeks.

### Endochondral bone formation

**Fig. 6:**
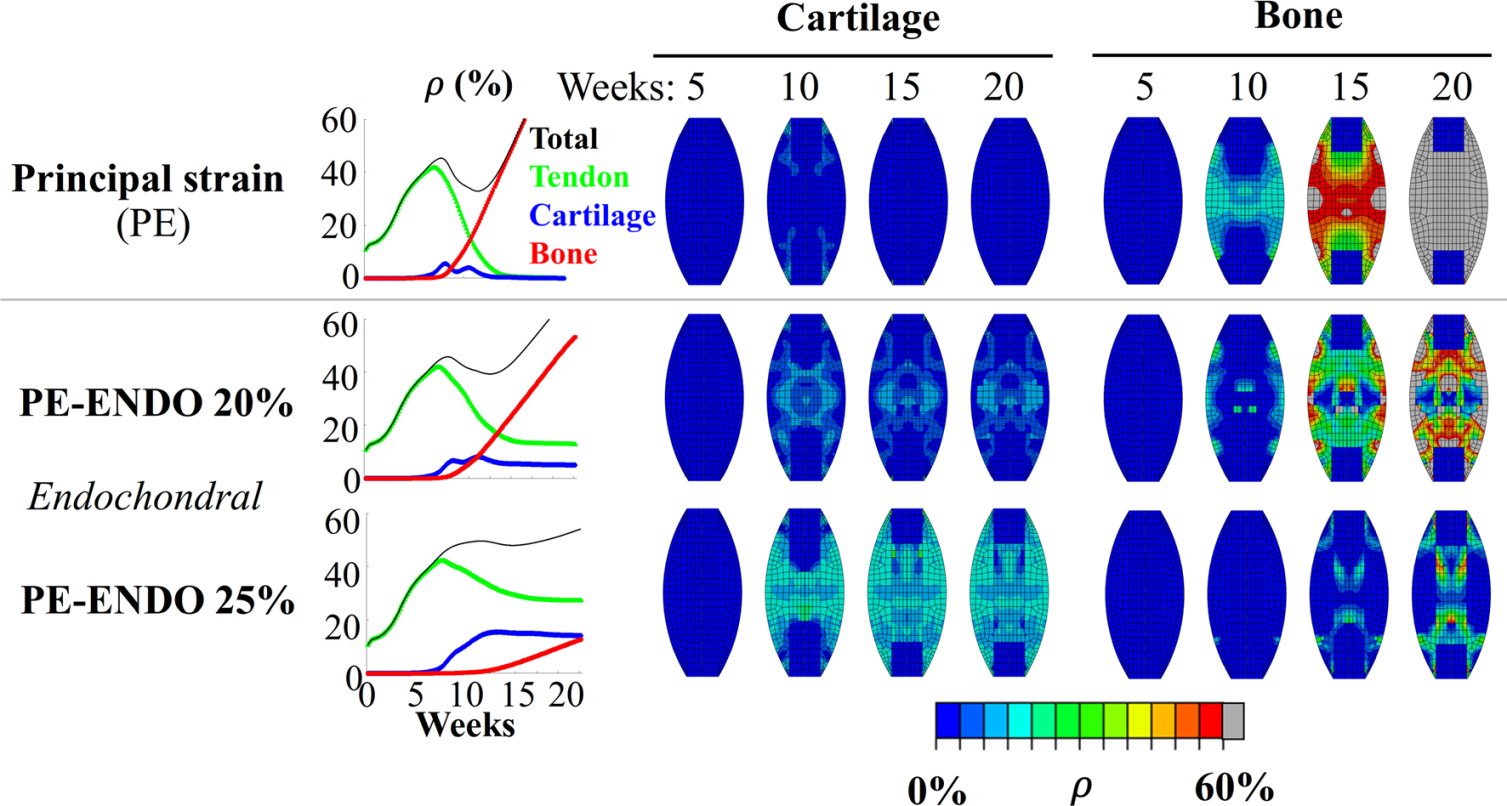
Effect of limiting bone formation to the endochondral pathway for the principal strain (PE) stimulus. Spatiotemporal predictions of tendon-, cartilage- and bone-like tissue density (*ρ*) throughout 20 weeks of healing is depicted

Exploring the effect of adding the requirement that bone-like tissue could only form through the endochondral pathway in the principal strain model (PE-ENDO 20% and 25%) decreased the predicted bone-like tissue content. Instead, more persistent tendon- and cartilage-like tissue formation was predicted, compared to the default principal strain model (PE) that did not limit bone-like tissue formation to the endochondral pathway (Fig. 6).

### Parameter sensitivity of the oxygen framework

**Fig. 7:**
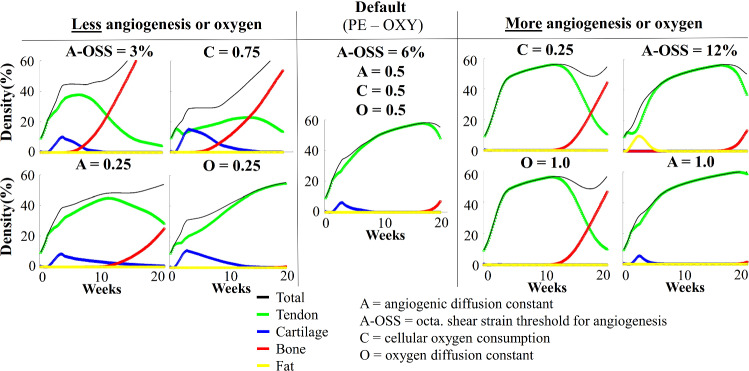
Parameter sensitivity analysis for the principal strain and oxygen framework (PE-OXY). The temporal evolution of the tendon-, cartilage-, bone- and fat-like tissue density, throughout the first 20 weeks of healing

The parameter sensitivity study for the principal strain and oxygen framework (PE-OXY) showed large variations in the predictions of temporal tendon-, cartilage-, bone- and fat-like tissue formation throughout the 20 weeks of healing (Fig. 7). Parameter perturbations that increased the process of angiogenesis or oxygen concentration (C = 0.25, A-OSS = 12%, O = 1.0, A = 1.0), generally predicted decreased formation of cartilage, whereas all perturbations that created more hypoxic conditions (C = 0.75, A-OSS = 3%, O = 0.25, A = 0.25), predicted increased formation of cartilage. Increased bone-like tissue content was predicted using different parameter perturbations that both increased and decreased angiogenesis and oxygen levels. Fat tissue was only predicted to form in one simulation case (A-OSS = 12%).

**Fig. 8:**
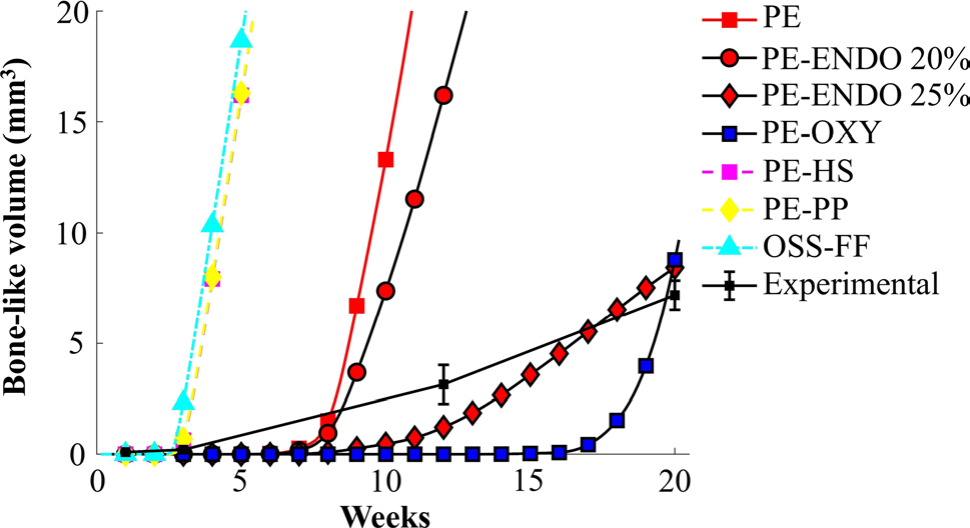
Temporal evolution of the bone volume for the different biophysical stimuli versus in-house experimental data (mean ± standard deviation, N = 3/time point) from healing rat Achilles tendon, subjected to free cage activity (Pierantoni et al. 2022). Experimental data were assessed at 1, 3, 12 and 20 weeks of healing

The temporal evolution of the volume of bone-like tissue in the healing callus was compared to in-house quantitative measurements of the total volume of bone-like tissue in healing rat Achilles tendon subjected to free cage activity at 1, 3, 12 and 20 weeks of healing (N = 3/time point) (Fig. 8) (Pierantoni et al. 2022). Most of the mechanobiological algorithms overpredicted the bone volume grossly, whereas the algorithms based on principal strain combined with oxygen (PE-OXY) or the principal strain combined with endochondral (PE-ENDO 25%) pathway mostly predicted the experimentally observed bone formation after 20 weeks of healing.

## Discussion

In this study, we present the development of the first computational framework investigating mechanobiological processes regulating formation of multiple tissue types during tendon healing. Specifically, we incorporated mechanical, cellular, angiogenesis and oxygen-related stimuli to predict heterogeneous tissue production, organization and mechanical properties during tendon healing. Different biophysical stimuli, e.g., principal strain, hydrostatic stress, pore pressure, octahedral shear strain and fluid flow, angiogenesis and oxygen concentration were considered, according to earlier work in bone healing (Burke and Kelly 2012; Isaksson et al. 2006a, 2008). The different biophysical stimuli displayed the capability to reproduce different experimental observations of spatial and temporal evolution of tendon, fat-, cartilage- and bone-like tissue during tendon healing in small animal studies.

Overall, the different biophysical stimuli predicted tissue production pathways roughly similar to experimental observations with predominantly tendon production throughout the first month of healing, followed by formation of cartilage-like regions and eventually predicting significant formation of bone-like regions. Tendon production was higher in the periphery since cell infiltration from the extrinsic compartment allowed early tendon production in the periphery, whereas the tendon core (lacking cells) could not produce tissue. However, several of the biophysical stimuli predicted too fast and too much bone formation (Fig. 8).

Temporally, one experimental study showed a decrease in tendon phenotype (less collagen type 1) and increase in cartilage-like phenotype (more collagen type 2) throughout 17 weeks of healing (da Silva et al. 2020). This exemplifies the decrease in tendon-like phenotype throughout time, as observed in most of our healing simulations. Yet, the experimental data also imply long-term presence of cartilage-like regions, and it was also shown that bone-like regions are surrounded by cartilage-like regions (Darrieutort-Laffite et al. 2019). Only the models with the principal strain and hydrostatic stress (PE-HS) or pore pressure (PE-PP) stimuli, and the principal strain with endochondral bone formation models (PE-ENDO), were able to predict long-term presence of cartilage.

Spatially, the different biophysical stimuli predicted rather small areas of cartilage-like tissue formation. This agrees qualitatively with experimental studies, as most studies observe rather small concentrated patches of proteoglycans (da Silva et al. 2020; Howell et al. 2017; Korntner et al. 2017; Misir et al. 2019). Different studies found cartilage-like staining (Korntner et al. 2017) and cells (Khayyeri et al. 2020) near the tendon stump. Howell et al. (2017) observed stronger proteoglycan staining toward the periphery and Khayyeri et al. (2020) also described more isolated islands of cartilage-like cells throughout the healing callus. Most of these spatial findings were predicted using the different algorithms.

In terms of bone-like tissue formation, experimental studies identified bone-like regions by 5–16 weeks of healing (Asai et al. 2014; Chen et al. 2017; Howell et al. 2017; Huber et al. 2020; Lin et al. 2010; Zhang et al. 2016). In alignment with these experimental results, the octahedral shear strain and fluid flow (OSS-FF), principal strain combined with hydrostatic stress (PE-HS) and pore pressure (PE-PP) algorithms predicted bone formation as early as four weeks of healing. Also, our models predicted large bone volumes in the whole callus, as observed experimentally at 15 (Sakabe et al. 2018) and 16 weeks (Hsieh et al. 2016) (Fig. 1). Yet, also the experimental studies show high variations in the locations and sizes of the different bone-like regions, and more experimental data would be valuable for further development and validation of the computational framework.

Throughout our study, we observed a high sensitivity of the healing predictions to the chosen parameters. One example is the production rate of tendon for the different stimuli. The principal strain stimulus, with (PE-OXY) or without oxygen (PE), predicted tendon formation according to the strain-regulated production law with a maximum production rate of (2%/day), similar to our recent healing frameworks (Notermans et al. 2022, 2021b). However, a constant production rate of 2%/day was used for the older mechanoregulatory models (PE-HS, PE-PP, OSS-FF). Consequently, the tendon density and stiffness evolved quicker in the older mechanoregulatory models, compared to the principal strain (PE) and oxygen (PE-OXY) stimulus, which were implemented more similarly to our recently developed healing framework. The slower recovery of stiffness for the strain (and oxygen) stimulus also contributed to slower evolution of bone production, since it took a longer time for strains to drop below the threshold value for bone formation (2%). The effect of the production rates is also reflected in the quantitative comparison of the temporal evolution of the total bone volume predicted in our frameworks with in-house experimental data (Fig. 8). Thus, most models overpredicted the volume of bone-like tissue throughout the simulation, when comparing to the in-house experimental data. Using the strain-regulated production law for tendon (PE, PE-OXY), particularly in combination with allowing only endochondral bone formation (PE-ENDO), predicted bone formation at a more similar rate to the experimental data than the older mechanoregulatory models. Yet, the default principal strain and oxygen stimulus (PE-OXY) still underpredicted the experimentally observed bone formation, but as indicated by the parameter analysis (Fig. 8), the predicted bone formation varies greatly with a large number of unknown parameters. Note that quantitative bone volumes from experimental data (Fig. 8) include bone-like volumes in the intact tendon stumps. This probably means that the presented data overestimate the bone-like tissue volume inside the healing callus. Elaborate data for validation should be used to calibrate parameters, such as the production rates of the different tissue types. Even though many model parameters need further characterization and validation, the current framework clearly shows, as a proof of concept, that this computational framework can be an important tool in understanding tendon mechanobiology during healing. Particularly, when trying to understand mechanobiological mechanisms of tissue differentiation or the formation of different tissue types during tendon healing.

We explored the effect of allowing only endochondral bone formation in combination with the principal strain modulus (PE-ENDO), as this has been proposed to be the main pathway of heterotopic ossification during Achilles tendon healing (Lin et al. 2010). Interestingly, this led to an increased production and persistence of tendon and cartilage, whereas it limited the predicted bone formation, compared to the default strain model. These results agreed better with the quantitative bone volumes in Fig. 8. Additionally, these models predicted experimental observations of long-term (> 15 weeks) presence of cartilage-like tissue (da Silva et al. 2020) in coexistence with more isolated islands of bone-like tissue (Darrieutort-Laffite et al. 2019), instead of a fully ossified callus without any cartilage-like tissue present. This last result highlights that modeling the endochondral bone formation process may be critical to predict reasonable cartilage- and bone-like tissue formation during tendon healing.

We also investigated a mechanobiological algorithm that combined mechanoregulation with predictions of oxygen and angiogenesis (PE-OXY) (Burke and Kelly 2012). In short, this model considered the ingrowth of blood vessels which provides oxygen to the healing callus from the periphery into the hypoxic tendon core (Online Resource 5). This framework shifted the cartilage-like tissue formation to the tendon core, as it is deprived of oxygen during early healing and cartilage production occurs under hypoxic conditions (Lin et al. 2010). On the other hand, blood vessel formation (through angiogenesis) has been found in bone-like areas in tendon (Darrieutort-Laffite et al. 2019). The default parameters used for the principal strain and oxygen framework (PE-OXY) predicted bone formation at a late stage of healing (> 15 weeks) and potentially underpredicted the amount of bone formation observed in experimental data. Yet, the parameter sensitivity analysis identified large variations in the temporal prediction of fat-, cartilage- and bone-like tissue formation with changes in different parameters that remain uncertain. Experimental measurements of spatial and temporal evolution of oxygen concentrations and blood vessel formation will be important to enable validation of these frameworks, similar to the validation in the bone healing framework (Burke and Kelly 2012). Only one healing model (A-OSS = 12%) predicted the production of fat tissue, and fat production was predicted during the first weeks of healing. This agrees with Khayyeri et al. (2020) that found fat-like cells during the first weeks of healing. We implemented a principal strain threshold for fat-like tissue prediction of 25% and material properties that scaled all constitutive material properties by 0.5, compared to tendon. Both the strain threshold and material properties for the fat-like tissue lack experimental validation and should be addressed in the future.

One limitation of our current framework is that the experimental data for cartilage-like (proteoglycan and collagen staining) and bone-like tissue formation (X-rays or μCT) show multiple, unconnected, localized and discretized areas of chondrification and ossification. Yet, the finite element modeling approach uses continuum mechanics, which has the inherent effect that ossified areas are fully integrated and connected to adjacent tissue, whereas it may be possible that these ossified areas are not fully integrated in the surrounded matrix. Future in situ imaging techniques may characterize the heterogeneous strain distribution around ossifications and may quantify the loadbearing of these ossifications. This type of imaging experiments may be key to determine the role of these ossifications on overall tendon mechanics, heterogeneous mechanical stimuli and mechanisms of tendon failure.

In this study, we developed a mechanobiological tendon healing framework to predict tissue differentiation during tendon healing types based on mechanoregulatory schemes in literature. A wide range of biophysical stimuli, including purely mechanical stimuli but also cell infiltration, angiogenesis and oxygen concentration were explored to govern the formation of tendon-, fat-, cartilage- and bone-like tissue throughout the first months of tendon healing. Different biophysical stimuli captured specific aspects of experimentally observed features. Specifically, we predicted experimental observations of heterogeneous tissue formation and showed that mechanobiology may play a role in governing tissue formation and tissue differentiation during healing. This study provides the first numerical tool to investigate mechanobiological mechanisms governing the formation of tendon and other tissue types during tendon healing. Further development and validation of this model are necessary when more spatial and temporal experimental data are available, yet this framework can aid in designing better rehabilitation protocols after tendon rupture.

## Supplementary Information

Below is the link to the electronic supplementary material.

Supplementary file1 (DOCX 2041 KB)
