# Supplementary material for: Predicting the formation of different tissue types during Achilles tendon healing using mechanoregulated and oxygen-regulated frameworks
Source: Biomech Model Mechanobiol. 2022 Dec 21;22(2):655–67. doi: 10.1007/s10237-022-01672-4 (PMC10097799; doi:10.1007/s10237-022-01672-4)
Supplement: Supplementary file 1 — Supplementary file1 (DOCX 2041 KB) [file 10237_2022_1672_MOESM1_ESM.docx]

**Predicting the formation of different tissue types during Achilles tendon healing using mechano-regulated and oxygen-regulated frameworks**

Thomas Notermans, Hanna Isaksson

Department of Biomedical Engineering, Lund University, Lund, Sweden

**-------------------------------------------------------------------------------------------------**

**SUPPLEMENTAL MATERIAL**

*Biomechanics and modeling in mechanobiology*

**
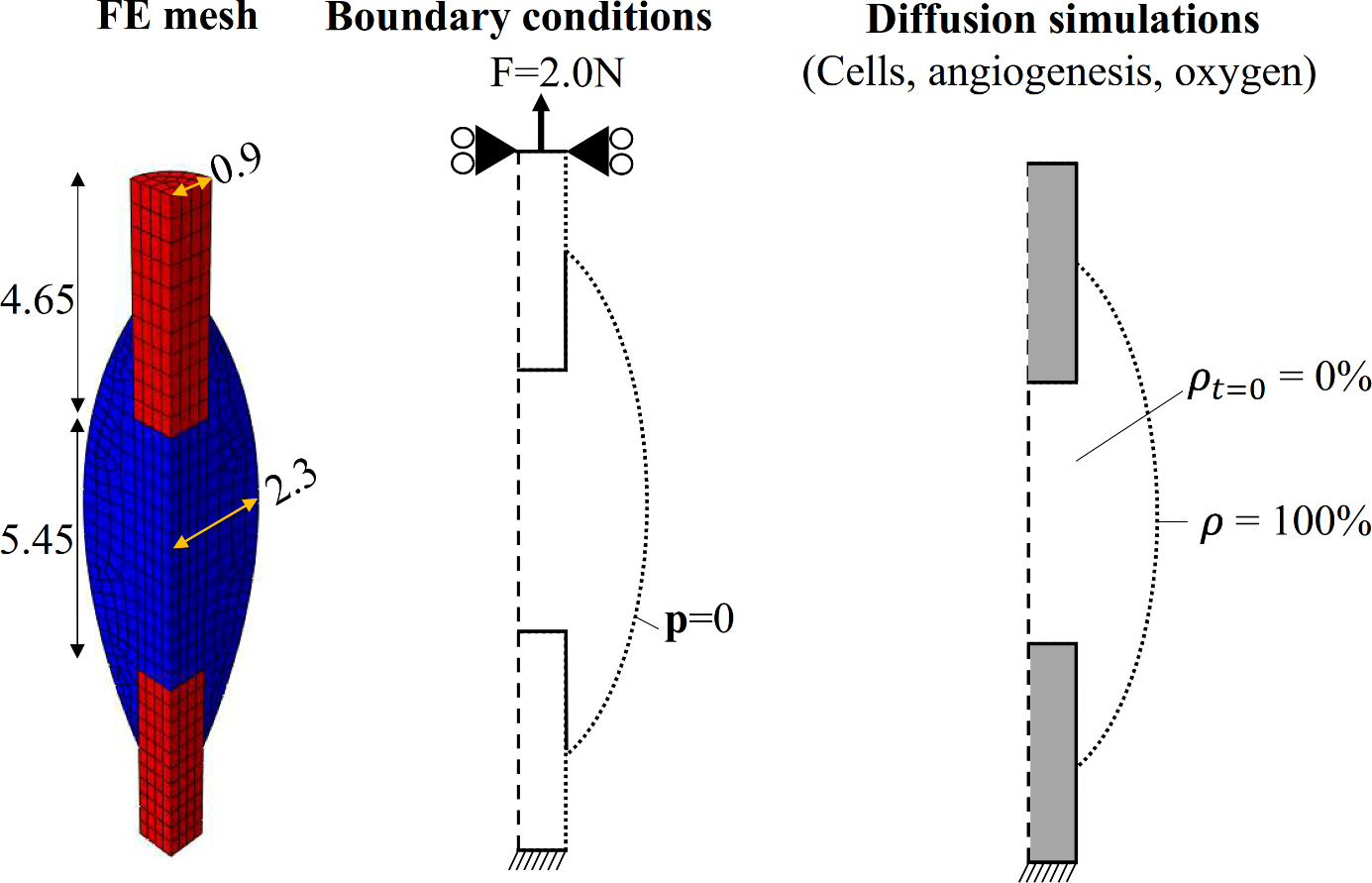
**

**Online Resource 1:** Schematic overview of the tendon mesh (dimensions in mm, from Khayyeri et al. 2020) and boundary conditions. The FE-model consist of a quarter-cylindrical geometry with symmetric boundary conditions applied. In the mechanical simulations, bottom end was clamped, and zero nodal pore pressure was prescribed on the external surface, and the force was applied on the top end of the tendon. The maximum force applied to the tendon was 2.0N. In the diffusion simulations that were used for modeling cell infiltration, angiogenesis and oxygen diffusion, their respective initial concentrations were set to 100% on the external surface of the callus, and there was no diffusion into the tendon stumps. During each iteration of the framework, diffusion for angiogenesis was allowed in elements with less than 6% octahedral shear strain.


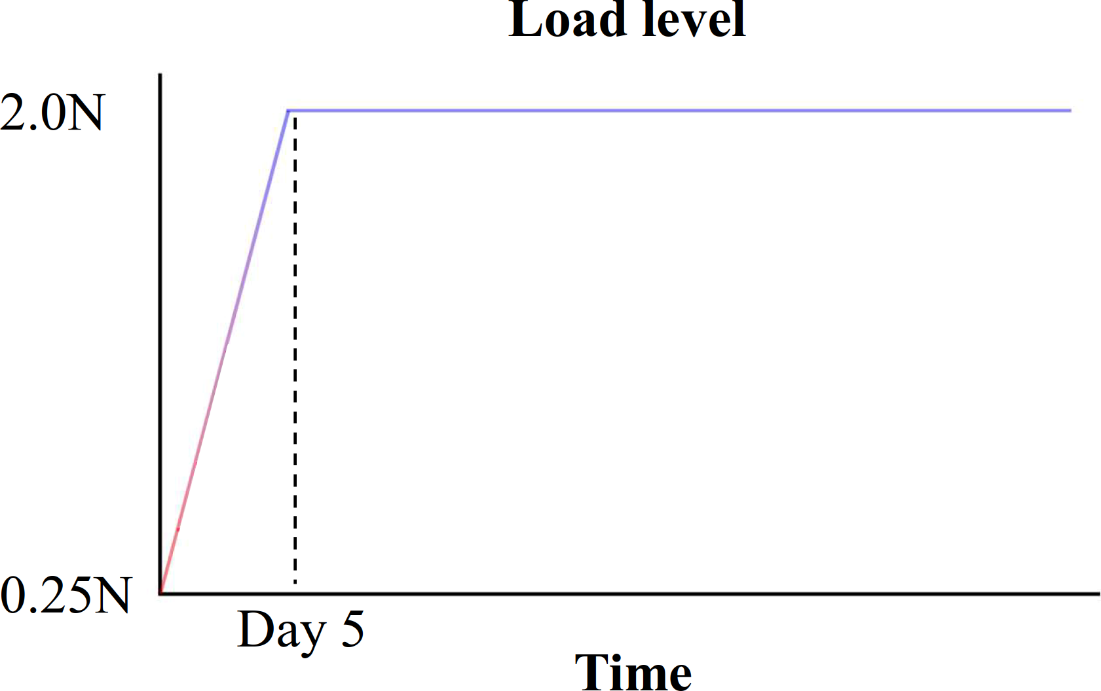


**Online Resource 2:** Applied force to the healing tendon throughout 20 weeks of healing. A force of 2.0N was used to mimic full physiological leading. Since the tendon is extremely weak at the first iteration of healing, the force is incrementally increased from 0.25N at the first iteration to reach 2.0N within 5 iterations (or days) of healing, similar to previous implementations (Notermans et al. 2022; Notermans et al. 2021b) .


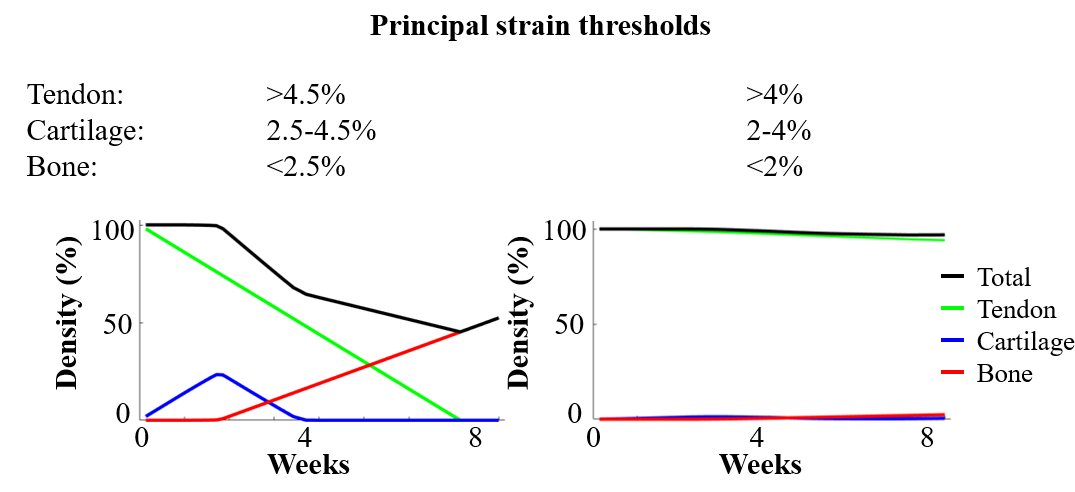


**Online Resource 3:** Identification of principal strain thresholds using remodeling in intact tendon, loaded with 2.0N. The intact tendon had the same length as the healing tendon geometry and intact mechanical properties were obtained from earlier work (Notermans et al. 2019). The daily production rate for tendon was 0%, and 2% for cartilage and bone formation. The principal strain thresholds (Tendon: >4%, Cartilage: 2-4%, Bone: <2%) resulted in no predicted cartilage and bone formation in a normal intact tendon.

**
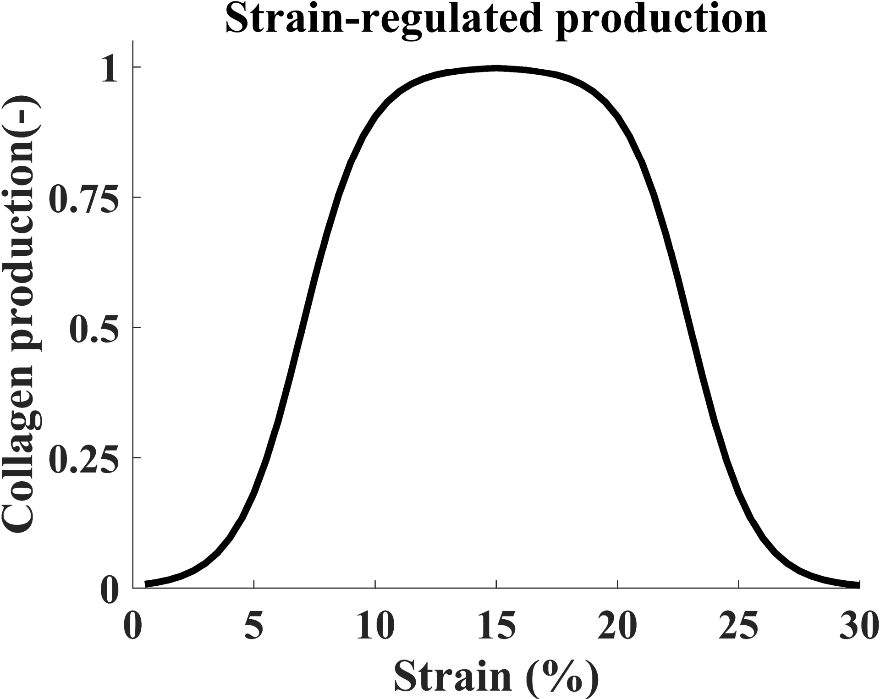
**

**Online Resource 4:** Strain magnitude-dependent scaling factor for the tendon tissue production rate of 2%/day, according to earlier work (Notermans et al. 2022; Notermans et al. 2021b). This production law was implemented in the principal strain with (PE-OXY) and without oxygen (PE) algorithm.


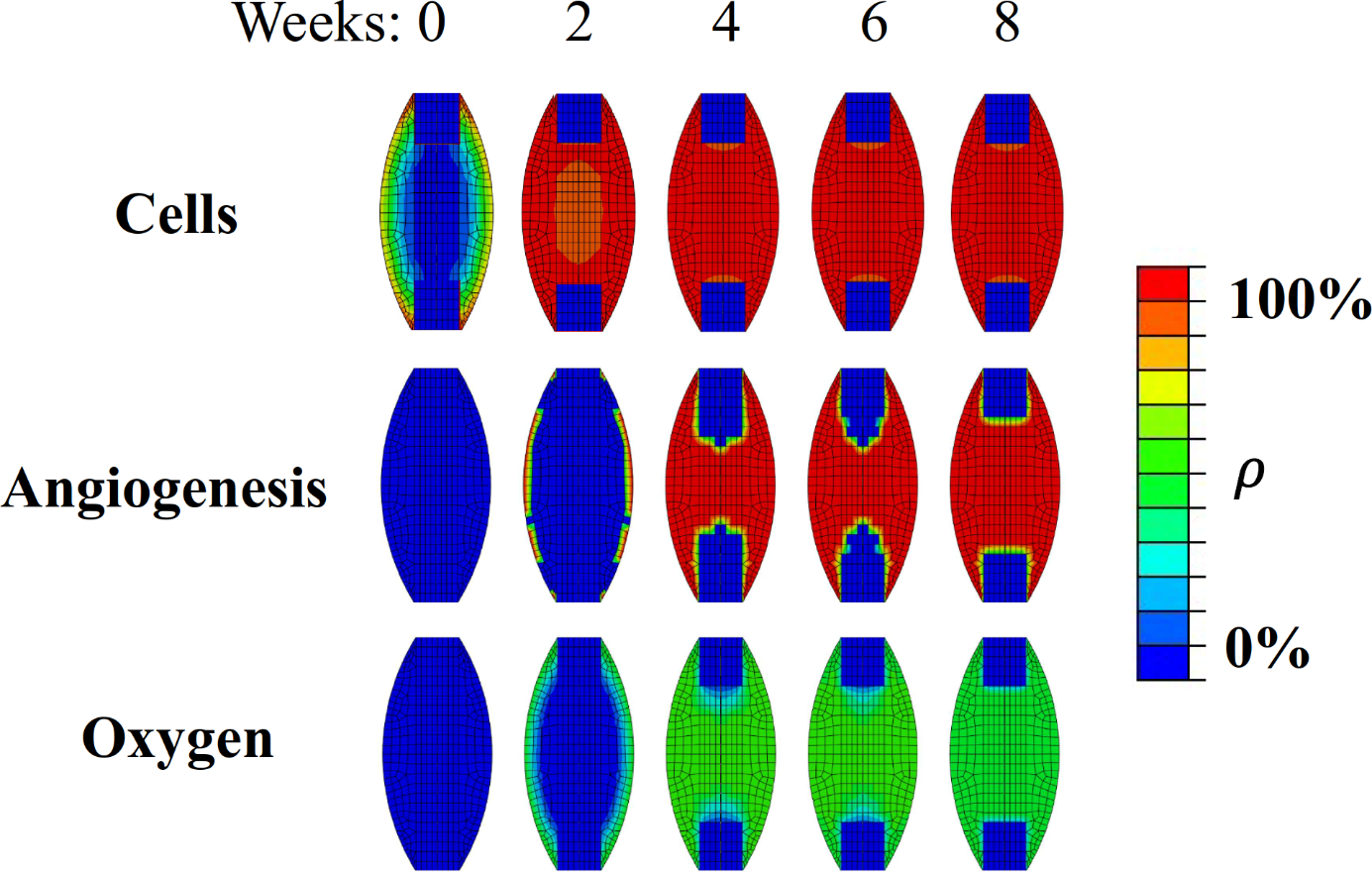


**Online Resource 5:** Spatio-temporal evolution of cells, angiogenesis and oxygen concentration in the principal strain and oxygen framework (PE-OXY). Note that the cell infiltration was not mechanoregulated, whereas angiogenesis was only allowed in elements with less than 6% octahedral shear strain, and that cells could consume oxygen. All healing simulations utilized cell diffusion, which mimicked infiltration of extrinsic cells into the empty callus, with a rate defined to predict that the whole callus was filled with cells after two weeks. The principal strain and oxygen framework (PE-OXY) included spatio-temporal evolution of angiogenesis (blood vessel formation) and oxygen levels. The spatial maps displayed the gradual infiltration of angiogenesis and oxygen saturation from the periphery of the callus into the callus core, within four weeks of healing. The angiogenesis and oxygen levels saturated (100% angiogenesis and oxygen levels) in the whole callus within the first 8 weeks of healing.
